# *PdMFS1* Transporter Contributes to *Penicilliun digitatum* Fungicide Resistance and Fungal Virulence during Citrus Fruit Infection

**DOI:** 10.3390/jof5040100

**Published:** 2019-10-18

**Authors:** Marta de Ramón-Carbonell, Mario López-Pérez, Luis González-Candelas, Paloma Sánchez-Torres

**Affiliations:** 1 Instituto Valenciano de Investigaciones Agrarias (IVIA), Centro de Protección Vegetal y Biotecnología, Moncada, 46113 Valencia, Spain; mardera2000@yahoo.es; 2 Instituto de Agroquímica y Tecnología de Alimentos (IATA-CSIC), C. Catedrático Agustín Escardino 7, Paterna, 46980 Valencia, Spain; mario.lopezp@umh.es (M.L.-P.); lgonzalez@iata.csic.es (L.G.-C.)

**Keywords:** citrus, fungicide resistance, MFS transporter, *Penicillium digitatum*, postharvest, virulence

## Abstract

A new *Penicillium digitatum* major facilitator superfamily (MFS) transporter (*PdMFS1*) was identified and functionally characterized in order to shed more light on the mechanisms underlying fungicide resistance. *PdMFS1* can play an important role in the intensification of resistance to fungicides normally used in *P. digitatum* postharvest treatments. In the *PdMFS1* disrupted mutants, a slight effect in response to chemical fungicides was observed, but fungicide sensitivity was highly affected in the overexpression mutants which became resistant to wide range of chemical fungicides. Moreover, *P. digitatum* knock-out mutants exhibited a lower rate of fungal virulence when infected oranges were stored at 20 °C. Disease symptoms were higher in the *PdMFS1* overexpression mutants coming from the low-virulent *P. digitatum* parental strain. In addition, the gene expression analysis showed an induction of *PdMFS1* transcription in all overexpression mutants regardless from which progenitor came from, and four-time intensification of the parental wild type strain during citrus infection reinforcing *PdMFS1* role in fungal virulence. The *P. digitatum* MFS transporter *PdMFS1* contributes not only to the acquisition of wide range of fungicide resistance but also in fungal virulence during citrus infection.

## 1. Introduction

*Penicillium digitatum* (Pers.: Fr) Sacc., is responsible for green mold, the most prevalent postharvest disease of citrus fruits [1]. Currently, control of this fungus is carried out by synthetic fungicides such as sterol demethylation inhibitors (DMIs), *O*-phenylphenol, or thiabendazole [2]. However, the continuous use of chemicals for preventing fungal diseases, has limited the efficacy and useful lifetime of fungicides. The emergence of fungal pathogens resistant to chemical compounds and the negative impact of fungicides on human health have warranted significant research attention in order to develop more effective control strategies. 

Knowledge of the mechanisms of fungicide resistance has been extensively explored in recent years [3,4,5]. Among all the mechanisms described, the overexpression of efflux pumps of two classes of transporter proteins that included the ATP-binding cassette (ABC) or the major facilitator superfamily (MFS) transporters has been of greater interest since they can confer resistance towards many unrelated toxic compounds through active transport out of the cell, resulting in decreased intracellular drug concentrations [6]. 

MFS transporters are included in the group of secondary active transporters that can transport substances in reaction to ion gradients. MFS transporters can mediate antiport, uniport, and symportation of different products [7]. Many of these MFS transporters are relevant for transporting small molecules in response to ion gradients or molecules that function as a drug that regulate microorganism growth under stress conditions affecting membrane potential and internal pH [8]. In addition, these transporters have little substrate affinity contributing in the transfer of wide range of substrates and, therefore, might play a function in sensitivity to different compounds.

In plant pathogenic fungi, the MFS transporters are able to pump off fungal toxins that contribute on the increase of fungal aggressiveness to host plants [9,10,11,12]. Furthermore, overexpression of MFS transporters could confer resistance to wide range of chemical drugs at the same time, thus acquiring the fungus a multidrug resistance (MDR) phenotype [8,13].

The effect on toxin efflux and fungicide sensitivity has been shown in different fungal MFS transporters. For instance, the elimination of the *Cercospora nicotianae* MFS transporter reduced cercosporin toxin [10]. In *Botrytis cinerea, BcMfs1* affected the sensitivity to camptothecin and cercosporin and DMI resistance [9] and *mfsM2* showed increased fungicide efflux activity [11]. The deletion of *MgMfs1* of *Mycosphaerella graminicola* affected resistance to strobilurin fungicides but not to other fungicides evaluated [12,13]. In *Zymoseptoria tritici*, the *MgMFS1* transporter contributed to the MDR phenotype [14]. Moreover, the *AaMFS19* MFS transporter was shown to play a role in resistance to oxidative stress and chemicals in the phytopathogenic fungus *Alternaria alternata* [15].

Recently, research conducted on *P. digitatum* reported two different MFS transporters *PdMfs1* and *Pdmfs2*. *PdMfs1* is apparently partially associated with imazalil-resistance and pathogenicity [16], while *Pdmfs2* seems to be involved in prochloraz sensitivity, sporulation, and fungal aggressiveness [17].

In this work, we identified a new *P. digitatum* MFS transporter that is relevant for acquisition of a wide range of fungicide resistance, and it is important for fungal virulence. *PdMFS1* characterization was carried out using gene deletion and gene overexpression in different *P. digitatum* strains and the evaluation of transcription profiling was also provided. Our results offer new insights into the molecular systems displayed by *P. digitatum* in the acquisition of fungicide resistance and higher virulence.

## 2. Materials and Methods

### 2.1. Microorganisms and Culture Conditions

Three different *Penicillium digitatum* strains were used: high virulent Pd1 resistant to imazalil, prochloraz, and thiabendazol among others; Pd27; and Pd149, both fungicide-sensitive and high and low virulent, respectively. All strains were already described in a previous work [4]. Fungal growth in solid and liquid media and conidia collection was carried out on potato dextrose agar (PDA; Liofilchem Laboratories, Teramo, Italy) or potato dextrose broth (PDB; Liofilchem Laboratories, Teramo, Italy) as reported before [18]. Cultures were grown at 25 °C with continuous light for one, two, or three days (liquid cultures), depending of the further use, or up to 1 week (agar media). Fungal spores were collected from 1 week old PDA plates by scraping them with a sterile spatula, and transferring them to sterile water. Spores were filtered, and counted with a haemocytometer, adjusting them to a desired final concentration.

*Escherichia coli* DH5α was used for cloning and plasmid storage. *E. coli* cultures were grown in LB (Luria Bertani) plates or LB liquid media amended with 100 μg/mL of kanamycin at 37 °C.

*Agrobacterium tumefaciens* C_58_C_1_ was employed for fungal transformation. Bacteria containing plasmid constructs was cultured in LB plates or LB liquid medium with 50 μg/mL Rifampicin and 100 μg/mL Kanamycin at 28 °C following the procedure already described [18].

Mature oranges (*Citrus sinensis* L. Osbeck) from the cultivars ‘Navelina’ or ‘Navelate’ not treated with fungicides, harvested from different orchards at IVIA in Moncada (Valencia, Spain), were used in this study.

### 2.2. Nucleic Acids Isolation

Genomic DNA was isolated from mycelium of *P. digitatum* grown in PDB as reported [19]. PCR amplicons obtained in this study were purified using Ultra Clean TM PCR Clean-up (MoBio, Solan Beach, CA, USA). Gene cloning was confirmed by DNA sequencing using the fluorescent chain-terminating dideoxynucleotide method [20]. 

RNA was extracted from frozen mycelium of *P. digitatum* grown in PDB using Trizol (Ambion Inc., Austin, TX, USA) following manufacturer’s recommendations. Total RNA during fruit infection was obtained from fruit peel discs as described previously [21]. 

### 2.3. Identification of PdMFS1

Primers M1-1 and M1-2 (Table S1) designed from the sequence of *P. digitatum* MFS transporter PDS09B02 [21] were used for identification of the genomic version by screening *P. digitatum* Pd1 DNA library as reported previously [18].

The analysis of the PdMFS1 protein domains were done using SMART (http://smart.embl-heidelberg.de). Protein sequence alignments were performed utilizing the Clustal W program [22] and Mega 7.0 program was used for the construction of phylogenetic tree with the neighbor-joining method [23].

### 2.4. PdMFS1 Gene Deletion and Gene Overexpression

Two different binary plasmids were constructed based on pRFHU2 and pRFHU vectors, for *PdMFS1* gene disruption and PdMFS1 overexpression following the same procedure described before [24].

For gene deletion, PdMFS1 promoter and terminator regions were fused to *hph* gene using pRFHU2. For gene overexpression, the complete *PdMFS1* gene including promoter and terminator was introduced in pRFHU plasmid following the strategy described previously [25].

C_58_C_1_ *Agrobacterium*-mediated transformation was performed as reported [19]. Mutants were selected from PDA plates amended with 100 μg/mL of hygromycin B as a selection media. 

Confirmation of gene knock-out and gene overexpression was done by PCR using genomic DNA. Analysis of gene copy number was done by qRT-PCR as described [18].

### 2.5. Virulence Tests

For in vivo assays, ‘Navelina’ mature oranges without chemical treatments were infected as described previously [26]. Infection experiments were performed using freshly harvested orange (*Citrus sinensis*) that were wounded at four places around the equatorial axis and infected with 10 μL of a spore suspension adjusted to 10^5^ conidia/mL. They were kept at 20 °C and 90% RH (Relative Humidity). Three replicates of five fruits each were carried out, and the infection experiments were done twice. As control, mock-inoculated fruits were used. Infection progression was measured as percentage of infected fruits (disease incidence) and diameter of macerated tissue (disease severity).

### 2.6. Fungicide Sensitivity Test

Increasing concentrations of four different chemicals, Imazalil (Textar I; Tecnidex), Prochloraz (Ascurit; Tecnidex), Philabuster (mixture of Imazalil and Pyrimethanil) (Decco Ibérica) (0, 0.5, 1, 2, 4, 8, and 10 μg/mL) and Thiabendazol (Textar 60 T; Tecnidex) (0, 0.5, 1, 2, 4, 8, and 10 μg/mL) were selected to evaluate fungicide sensitivity of *P. digitatum* strains. Each treatment was carried out using three replicas and two independent experiments. The measurements were performed in 96-well microtiter plates comparing growth at different fungicide concentrations with untransformed wild-type strain as a control assayed at the same time. The activity and efficacy of the different fungicides were evaluated following the protocol described before [25]. Sensitivity to different chemical compounds was measured as the average of the ratio between growth in presence of fungicides and the growth in absence of the chemicals expressed in percentage. 

### 2.7. Quantitative Real-Time PCR

PrimeScript™ RT reagent Kit (Takara Bio Inc., CA, USA) was used for synthesis of the first strand of cDNA in a 20 μl reaction, following the indications of the manufacturer. Quantitative PCR was done as reported [18].

Values obtained resulted from the average of three biological replicates and two independent experiments. For *PdMFS1* quantification, primers qM1F and qM1R were used, and as independent reference genes were selected β-tubulin (qTubF-qTubR), ribosomal 28S (q28SF-q28SR), and histone H3 (qH3F-qH3R) (Table S1). 

The software LightCycler 480 SW 1.5 (Roche Diagnostics, Madrid, Spain) was used for cycle point quantification. Primer melting temperature allowed the selection of each primer set for specific amplification. The Relative Gene Expression (‘RGE’) was carried out as previously described [27].

### 2.8. Statistical Analysis

Evaluation of significant differences for sensitivity to chemicals and infection progression data was done using analysis of variance (ANOVA) with SAS software (SAS Institute Inc., Cary, NC, USA) statistical package. Statistically significance was considered when *p* < 0.05 using Tukey’s test for the separation of means. 

## 3. Results

### 3.1. Analysis of PdMFS1

In a previous work, we constructed a cDNA library enriched in *P. digitatum* genes that were induced during citrus fruit infection using suppression subtractive hybridization (SSH) [21]. Among those *P. digitatum* genes that showed a significant differential expression during the infection, we found a cDNA (PDS09B02) that exhibited high homology to the MFS transporter family, and it was selected for functional characterization. 

Screening of the *P. digitatum* Pd1 genomic library allowed to obtain the *PdMFS1* genomic version that included exons and introns. Sequence evaluation showed that the genomic region contained an open reading frame of 2327 bp, with two introns of 79 and 64 bp, placed at positions 2298..2469, 2548..4374, and 4438..4625 of the coding region, and the deduced protein encoded 728 amino acids. This gene was named *PdMFS1*, and encodes a major facilitator superfamily transporter in *P. digitatum*. The presence of introns and exons in the *PdMFS1* gene was done by sequencing the full cDNA fragment and compared to the genomic version. The *PdMFS1* gene nucleotide sequence is accessible in GenBank as GU124565. 

The nucleotide sequence of the *PdMFS1* gene was compared in the three isolates of *P. digitatum* strains used (Pd1, P27, and Pd149). No differences were found either in the promoter region or in the coding region.

**Figure 1:**
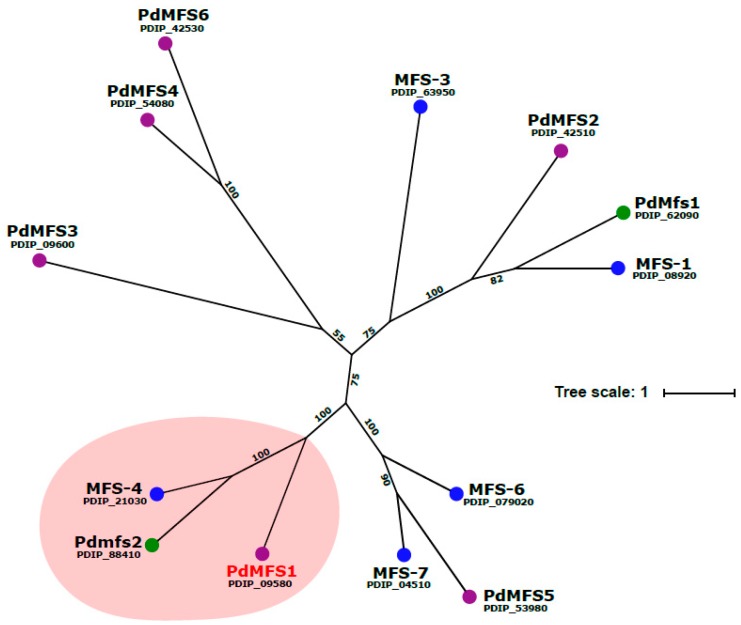
Unrooted Maximum Likelihood phylogenetic tree of selected *P. digitatum* major facilitator superfamily (MFS) transporters. PdMFS1, the new MFS transporter described in this study, is highlighted in red. Values indicate the number of times (in percent) that each branch topology was found during bootstrap analysis. The National Center for Biotechnology Information (NCBI) accession number is indicated below the name of each transporter.

PdMFS1 showed characteristic DNA-binding motifs, particularly a domain with high identity to MFS transporters. The software TMPRED (Swiss Institute of Bioinformatics) was used for hydropathic analysis that showed that *PdMFS1* has 12 putative transmembrane domains. Alignment of *PdMFS1* sequence with other *P. digitatum* MFS transporters sequences available at the National Center for Biotechnology Information (NCBI) exposed a *PdMFS1* cluster close to the *Pdmfs2 P. digitatum* MFS transporter [17] and MFS-4 [28], and far away from *P. digitatum PdMfs1* in terms of homology and location [16] (Figure 1).

### 3.2. Generation of the PdMFS1 Mutants

**Figure 2:**
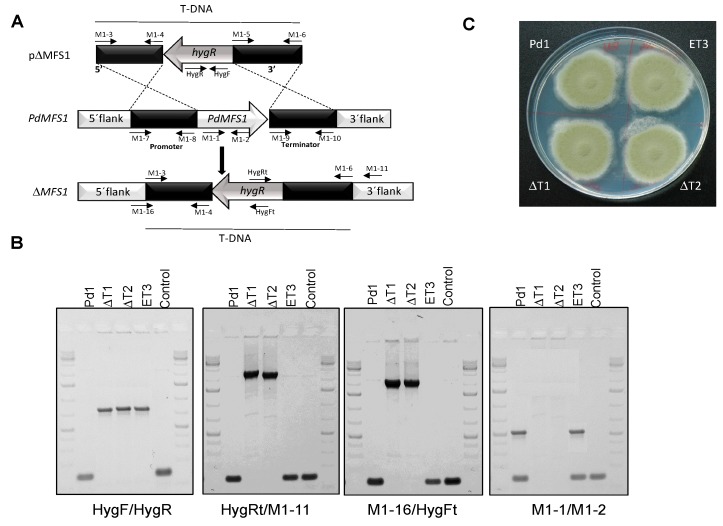
(**A**) Diagram of wild-type locus and the *PdMFS1* replacement with the HygR selectable marker from pΔMFS1 by homologous recombination to generate the ΔMFS1 mutants; (**B**) Polymerase chain reaction (PCR) evaluation of the wild-type Pd1 strain, two Δ*PdMFS1* null mutants (ΔT1, ΔT2) and the respective ectopic mutant (ET3) with analytic primers. (**C**) Phenotypical traits and conidiation. Fungal strains grown in potato dextrose agar (PDA) plates at 25 °C during six days.

In order to characterize *PdMFS1* function, gene deletion was performed in *P. digitatum* Pd1 from which the *PdMFS1* gene was obtained. The pΔMFS1 plasmid comprises a *PdMFS1* promoter region amplified using M1-7–M1-8 primers (Table S1), followed by the hygromycin B resistant cassette and a *PdMFS1* terminator region using M1-9–M-10 primers (Table S1). pΔMFS1 vector was used for *Agrobacterium tumefaciens*-mediated transformation in the *P. digitatum* Pd1 strain (fungicide resistant and high virulent) (Figure 2A). 

Single spore mutants were grown on selected media and then mutants were confirmed by PCR for the presence of the hygromcycin B resistance marker with primers HygF-HygR. Verification of the elimination of *PdMFS1* coding region was done by PCR in both flanks with primers HygRt/M1-11 and M1-16/HygFt. No amplification was observed in the wild-type (WT) strain and in ectopic transformants that consisted of Pd1 mutants containing pΔMFS1 plasmid but not inserted in locus, while null mutants showed an amplicon of 2.9 kb and 2.1 kb, respectively (Figure 2B). The PdMFS1 gene elimination in the null mutants was proven by the absence of amplicon when primers M1-1/M1-2 (Table S1) were used (Figure 2B). 

To avoid the presence of null mutants with additional T-DNA integrations, the DNA copy number was analyzed in 10 mutants by real-time quantitative PCR (qRT-PCR), using WT as control and as a reference the β-tubulin gene. Among all transformants, we selected for further analysis ΔT1 and ΔT2 containing only a single T-DNA integration and one ectopic mutant (ET3). Evaluation of phenotypic traits revealed no differences in growth or sporulation on PDA between the deletants (ΔT1 and ΔT2), the ectopic mutant (ET3), and the WT strain (Figure 2C).

### 3.3. Overexpression of PdMFS1 in Different P. digitatum Strains

**Figure 3:**
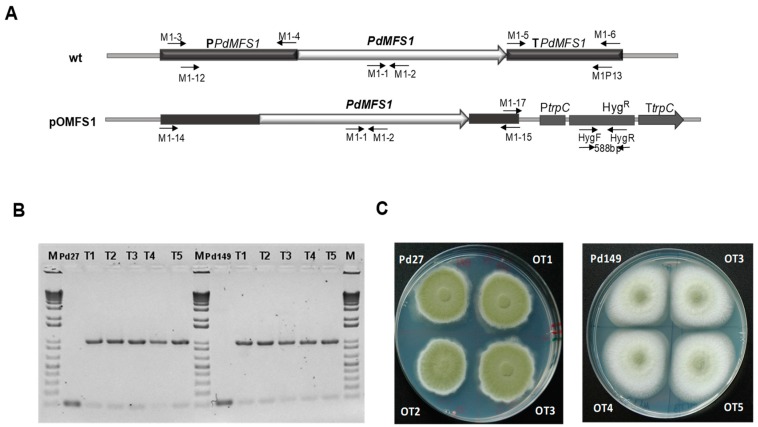
Construction and analysis of *P. digitatum* overexpression *PdMFS1* transformants. (**A**) Map of plasmid pOMFS1. Diagram of wild-type locus and the *PdMFS1* gene with the HygR selectable marker from pOMFS1 inserted elsewhere in the genome. Primers used in the construction of plasmid pOMFS1 and those used for the analysis of the transformants are shown. (**B**) Hygromycin polymerase chain reaction (PCR) analysis of the Pd27 wild type and Pd27-transformants, Pd149 wild type Pd149, and 149-transformants using primers HygF/HygR. Lanes correspond to WT: DNA from Pd27 strain, T1–T5: Pd27-overexpressed mutants and WT: DNA from Pd149 strain, T1–T5: Pd149-overexpression mutants. (**C**) Phenotypical features and conidiation. Fungal strains grown in PDA plates at 25 °C during six days.

To evaluate the effect of the MFS overexpression in *P. digitatum* strains sensitive to several fungicides, *P. digitatum* Pd27 and Pd149 strains (both fungicide sensitive and with high and low virulence, respectively) were transformed using *A. tumefaciens*-mediated transformation (ATMT) with pOMFS1 plasmid (Figure 3A). This plasmid is derived from pRFHU vector [24] in which the full *P. digitatum PdMFS1* coding sequence was introduced together with 1.2 kb of the promoter and 0.9 kb of the terminator (0.9 kb) using M1-12/M1-13 oligos (Figure 3A). Five transformants of each strain were confirmed by the presence of the hygromycin B gene using HygF/HygR oligos (Figure 3B). The 588 bp PCR amplicon was present in all transformants, but not in both Pd27 and Pd149 wild type strains. The T-DNA copy number was determined in all mutants from each strain by qRT-PCR as described above. Pd27 and Pd149 wild type strains were used as a control and β-tubulin was the reference gene. Of all the evaluated mutants, we chose OT1 and OT2 of Pd27 and OT3, and OT4 of Pd149 that contained one (OT1 and OT3) or two (OT2 and OT4) T-DNA copies integrated elsewhere in the genome (two or three copies of the *PdMFS1* gene, respectively). 

Evaluation of phenotypic features showed no differences between the mutants and their respective parental strains neither in growth nor in sporulation in PDA (Figure 3C).

### 3.4. Fungicide Sensitivity 

**Figure 4:**
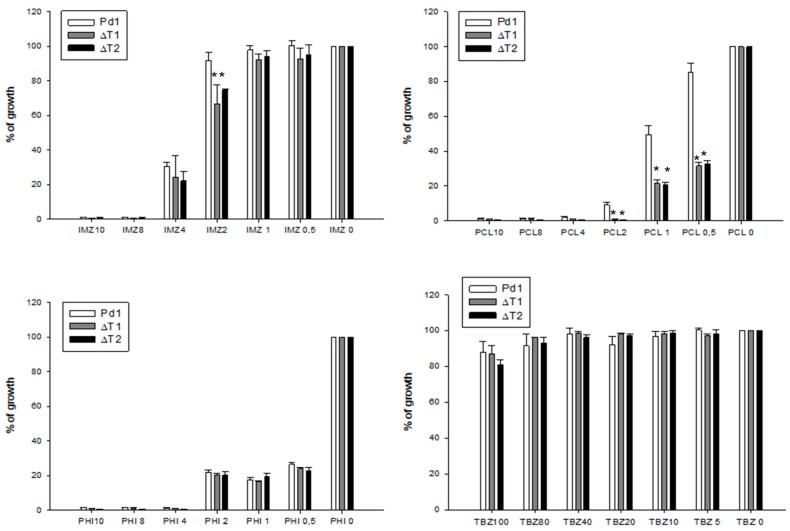
Evaluation of fungicide sensitivity in disruptant mutants compared to wild type Pd1. IMZ = imazalil, TBZ = Thiabendazol, PCL = Prochloraz, PHI = Philabuster. The fungicides concentration is expressed in μg/mL. Percentage of relative growth was calculated respect to the each strain grown without fungicide. Error bars represent standard deviation among three replicas. * Significant differences between treatments using Tukey’s test (*p* < 0.05).

Growth over 7 days at 25 °C of all selected *P. digitatum* strains (transformants and WT strains) in the presence of different concentration of fungicides showed no variation in fungicide sensitivity between transformants without the *PdMFS1* gene and WT Pd1 parental strain for IMZ, PHI (mixture of IMZ and pyrimethanil), and TBZ. However, when Prochloraz (PCL) fungicide was used, fungicide sensitivity increased in deletant mutants, entailing 25% growth versus almost 60% growth observed in Pd1 at 1μg/mL of PCL (Figure 4).

**Figure 5:**
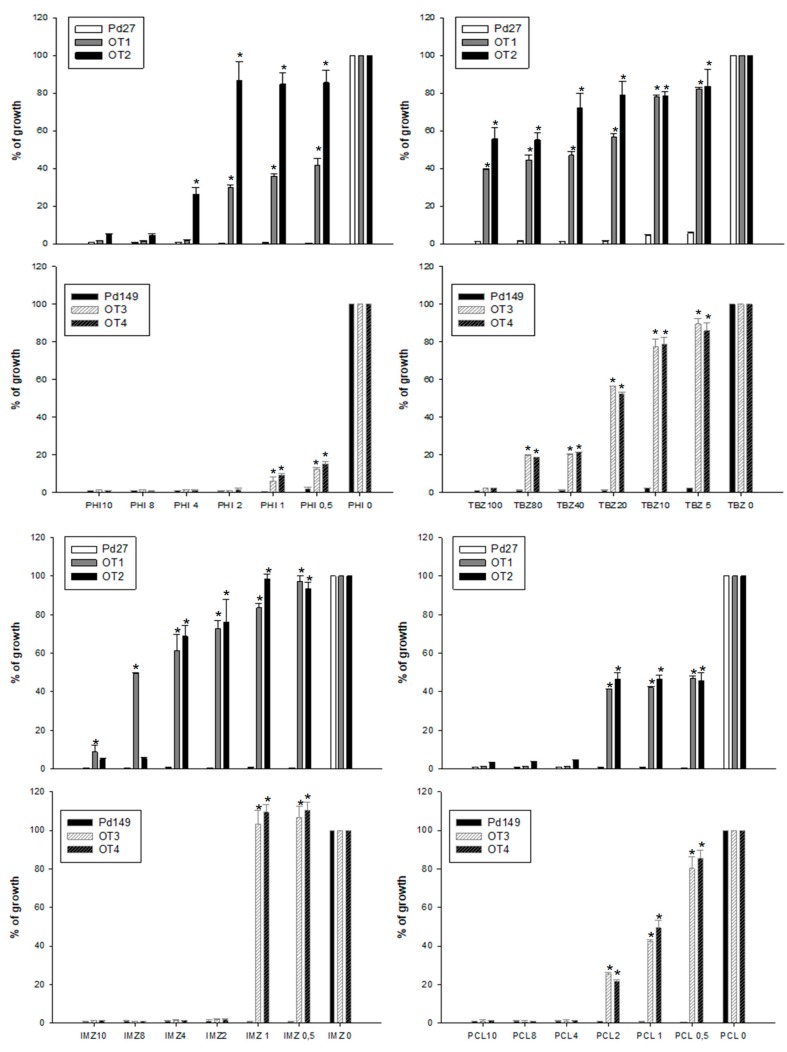
Evaluation of fungicide sensitivity in Pd27-(OT1, OT2) and Pd149-overexpression mutants (OT3, OT4) compared to both wild-type Pd27 and Pd149, respectively. PHI = Philabuster, TBZ = Thiabendazol, IMZ = imazalil, PCL = Prochloraz. The fungicides concentration is expressed in μg/mL. Percentage of relative growth was calculated respect to the each strain grown without fungicide. Error bars represent standard deviation among three replicas. * Significant differences between treatments using Tukey’s test (*p* < 0.05).

Conversely, all overexpression mutants (OT1 to OT4) regardless parental strain showed multiple effect on fungicide sensitivity. The rate of fungicide resistance was higher in Pd27 mutants than in Pd149 mutants, as was shown for IMZ and TBZ fungicide, while for PCL the intensification was similar (Figure 5). Pd27 transformants were more resistant to all fungicides evaluated compared to Pd149 transformants. Pd149 mutants only were resistant until 1μg/mL IMZ and the percentage of growth reached was always less than the rise for Pd27 mutants. It was also noticeable that the Pd27 transformants exhibited a constant rate of PCL fungicide resistance expressed as 40% of growth while Pd149 transformants showed a progressive decrease concomitant to fungicide increase concentration starting with 80% of growth to end to 20%.

### 3.5. Virulence Evaluation

In order to evaluate whether *PdMFS1* contributed to fungal pathogenicity/virulence, the evaluation of aggressiveness of the different *P. digitatum* mutants was done with infected orange fruits.

All strains used in this study were assayed, indicating disease incidence as a percentage of fruit infected and disease severity as area of macerated surface at different days post infection (dpi). 

**Figure 6:**
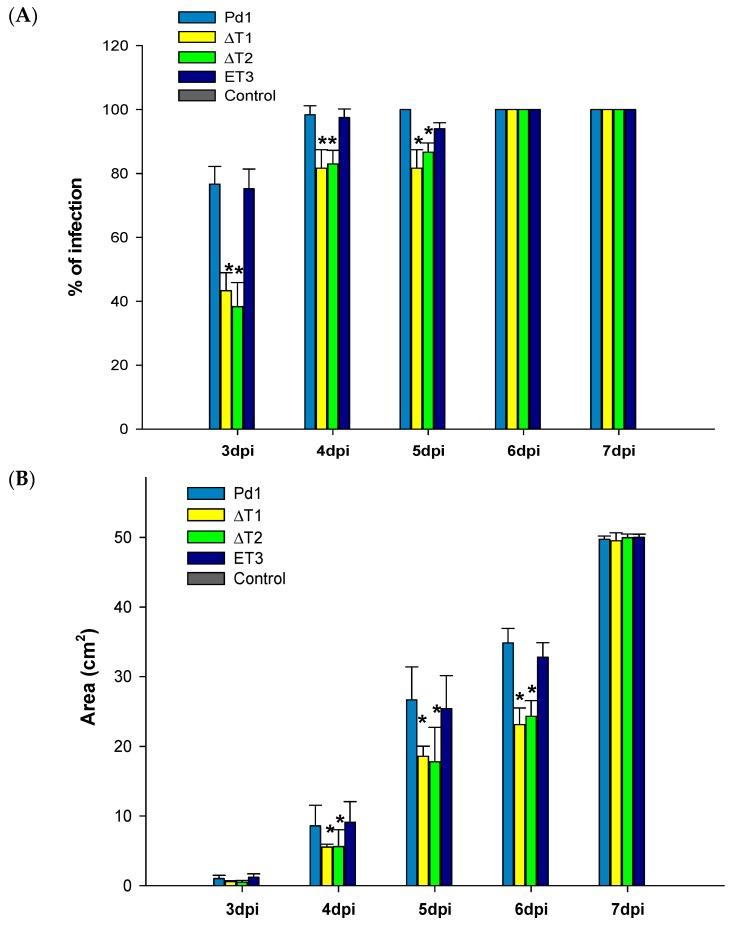
Analysis of fungal aggressiveness (**A**): disease incidence (%) and (**B**): disease severity (cm^2^). Virulence evaluation of Pd1, ectopic transformant ET3 and disruptant transformants (ΔT1, ΔT2). All are mean of two infection experiments. Control correspond to oranges mock inoculated. Error bars represent standard deviation. * Significant differences between treatments using Tukey’s test (*p* < 0.05) at each dpi.

*P. digitatum* ectopic strain ET3 infection progressed identical as the parental strain Pd1. However, the deletion of *PdMFS1* gene (∆T1 and ∆T2) resulted in a decrease in virulence, particularly during the first stages (3–4 dpi), as was shown on percentage of disease incidence (Figure 6A). The effect was more noticeable on disease severity than the one observed on disease incidence since the decrease was observed from 4–6dpi. (Figure 6B). The average of virulence reduction in the deletant mutants supposed a decrease between 20 and 40 of disease incidences and around 55–67 % of disease severity. At 7 dpi, no significant differences in the virulence were observed. 

**Figure 7:**
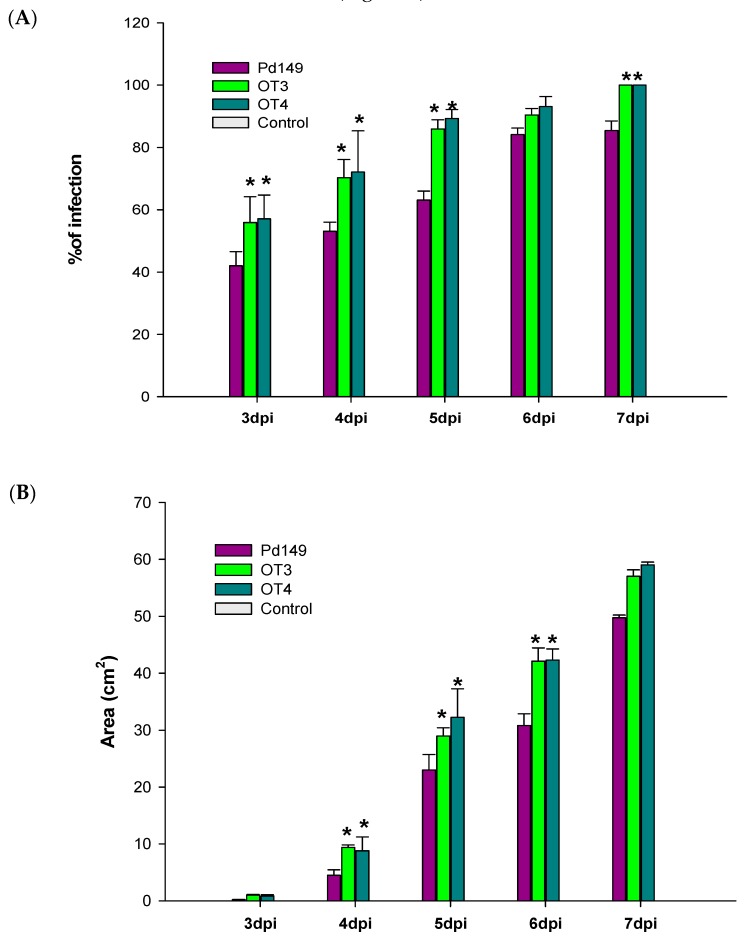
Evaluation of virulence as (**A**) disease incidence (%) and (**B**) disease severity (cm^2^). Virulence evaluation of Pd149 and the overexpression transformants (OT3, OT4). All are mean of three infection experiments. Control correspond to oranges mock inoculated. Error bars represent standard deviation. * Significant differences between treatments using Tukey’s test (*p* < 0.05) at each dpi.

Virulence analysis was also performed for overexpression mutants. Pd27 transformants did not show any differences when compared to parental strain Pd27 and fungal strains behaved similar in incidence and severity (Supplementary Figure S1). On the contrary, Pd149 transformants (OT3 and OT4) exhibited an increase in virulence in both disease incidence and disease severity, although rate of disease incidence did not reach that of Pd1 (Figure 7).

### 3.6. Expression Pattern of PdMFS1 

**Figure 8:**
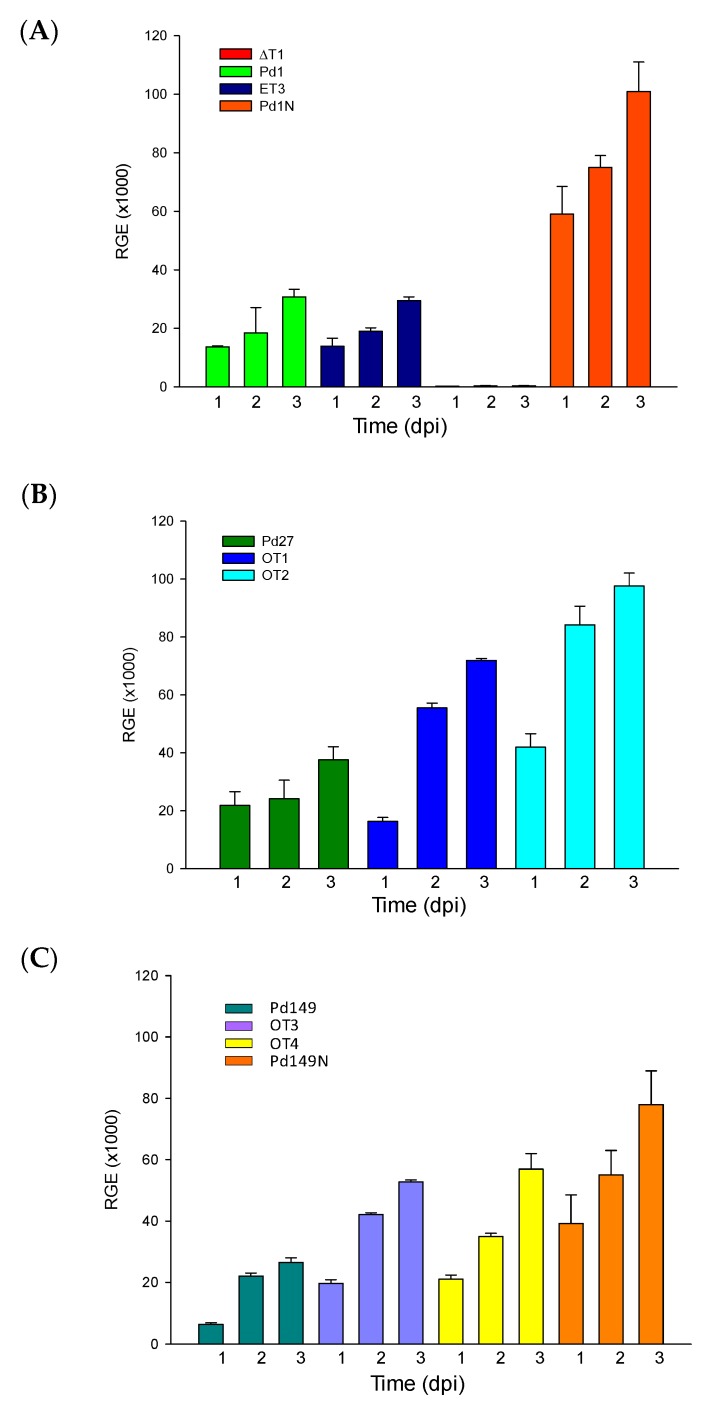
Analysis of *PdMFS1* relative gene expression (RGE). (**A**) Time course evaluation of gene expression of Pd1 and deletant mutants in PDB liquid culture at 25 °C (Pd1, ΔT1, ET3) and during orange infection (Pd1N). (**B**) Time course evaluation of gene expression of Pd27 and overexpression transformants in PDB liquid culture at 25 °C. (**C**) Time course evaluation of gene expression of Pd149 and overexpression mutants in PDB liquid culture at 25 °C (Pd149, OT3, OT4) and during orange infection (Pd149N). In all cases: 1, 2 and 3 correspond to 1, 2 and 3 dpi, respectively. The expression levels are relative to three reference genes: ribosomal 28S RNA, β-tubulin and histone H3. Error bars indicate standard deviations of three biological replicates.

Transcription levels of *PdMFS1* during axenic growth were the same in the WT-Pd1 as the ectopic mutant ET3, and gene elimination showed the absence of *PdMFS1* gene expression. Evaluation of gene transcription during orange infection of Pd1 strain showed that *PdMFS1* was increased during citrus fruit infection, reaching four times more gene transcription throughout the entire time assayed (Figure 8A). 

The transcription level of the *PdMFS1* gene was higher in all overexpressed mutants, regardless of the strain selected. Differences in the transcriptional rate were observed in OT1 compared to OT2, while no differences were observed for OT3 and OT4 (Figure 8B,C). Moreover, *PdMFS1* gene expression in Pd149, similarly to Pd1, exhibited up-regulation during infection, although in this case the rate was only two times higher than during axenic growth (Figure 8C).

## 4. Discussion

The continuous appearance of fungicide resistant strains questions the effectiveness of fungicides, especially after public concern about the impact of these chemical compounds on health. This fact underscores the need to develop new targets to achieve a more efficient control in citrus postharvest. In that sense, MFS transporters might be a good alternative since they could play a part in multidrug resistance in bacteria and fungi [29].

The current work contributes to the clarification of the role of *P. digitatum* MFS transporters regarding to fungicide resistance and fungal virulence. This study is focused on identification and functional evaluation of *PdMFS1*, a new major facilitator superfamily transporter gene from *P. digitatum. PdMFS1* exhibited a typical structure of MFS transporters containing a 12- transmembrane domain and showed a close phylogenetic relationship to one reported *P. digitatum* MFS transporter, *Pdmfs2* [17], and other *P. digitatum* MFS transporters apparently up-regulated in the presence of prochloraz [28].

The functional analysis showed that this new transporter, *PdMFS1*, is involved in prochloraz sensitivity since its deletion decreased of 50% fungal growth at 0.5–2 μg/mL PCL concentration. This might indicate that *PdMFS1* could pump prochloraz out of cells, as was shown for another *P. digitatum* MFS transporter (*Pdmfs2*) in a previous work [17], and in *MgMFS1* of *Z. tritici* [14]. Nevertheless, the effect of this transporter in fungicide resistance seems to be wider since the overexpression of *PdMFS1* transporter in two different fungicide sensitive strains (Pd27 and Pd149) resulted in a marked increase in resistance to prochloraz and to other fungicides frequently used in citrus postharvest such as imazalil, Philabuster (combination of imazalil and pyrimethanil), and thiabendazole. This result indicates that this transporter is capable of expelling a wide range of toxic products since these fungicides belong to different groups. *PdMFS1* overexpression increased the level of fungicide resistance in a more noticeable way in the Pd27 strain than the Pd149 strain. The level of resistance reached by Pd149 was lower for all the fungicides tested with the only exception of the prochloraz, where the rate was much more similar. The differences observed between the two strains (Pd27 and Pd149) tested for *PdMFS1* overexpression could be explained by the different growth or fitness of each strain. In fact, Pd27 is able to grow and infect much more effectively than the Pd149 strain.

This circumstance, together with the effect observed when *PdMFS1* was deleted, would indicate a predominant role in pumping out prochloraz and the ability to eliminate other toxic compounds. So far, the two other MFS transporters characterized in *P. digitatum* showed that *PdMfs1* is only associated with partial imazalil resistance [16], and Pdmfs2 appear to only partially contribute to prochloraz resistance [17]. Moreover, seven different *P. digitatum* MFS transporters were identified as being up-regulated in presence of prochloraz fungicide, indicating that the same function could be carried out for different genes of the same family [28].

The participation of fungicide sensitivity has been reported in other fungal MFS transporters, for instance, an MFS protein gene knockout in the *Trichoderma harzianum* fungus caused a loss of tolerance to fungicides [30]. Fungal mutants lacking the *A. alternata* MFS transporter *AaMFS19* display sensitivity towards different compounds [15]. In *B. cinerea,* the contribution of *BcMfs1* in the resistance of DMI fungicides was proven after elimination of the ABC transporter *BcatrD* and in the *BcMfs1* overexpressing strains [9,31], following the same pattern that was observed by *PdMFS1* for DMIs and TBZ fungicides

These results suggest that MFS transporters from different organisms present high complexity, and although MFS transporters belonged to the same family, could transport very diverse types of toxic compounds.

Analysis of gene expression confirmed that *PdMFS1* exhibited the same rate of gene transcription in both fungicide-resistant (Pd1) and -sensitive strains (Pd27 or Pd149) of *P. digitatum* during axenic growth, indicating that basic efflux mechanism in *PdMFS1* was present in all strains of *P. digitatum*. 

Even if the sensitivity of Δ*PdMFS1* to IMZ was slightly higher than that of the parental strain Pd1, it was still much more resistant than the one observed on DMI-sensitive strains Pd27 or Pd149. Therefore, *PdMFS1* could only take part in influencing the baseline sensitivity to different fungicides (DMIs or bezimidazoles), as happened for ABC-based MDR [32] and for *PMR1* (an ABC transporter gene of *P. digitatum*), but it could not be the major determinant in DMI resistance [33]. In all likelihood, IMZ resistance is mainly provided by overexpression of sterol demethylases genes (CYP51) [34,35,36], and benzimidazole resistance is acquired through beta-tubulin mutations [4]. In likelihood, the reason why the elimination of *PdMFS1* does not drastically increase the sensitivity to all fungicides is that in the absence of a specific MFS transporter, other transporters of the same family can replace their function. This effect has been observed when the *P. digitatum* sucrose transporter *PdSUT1* was deleted and the transcription of six different *P. digitatum* MFS transporters, including *PdMFS1,* was augmented [25].

By analyzing conidiation, we observed that neither deletion mutants nor overexpression mutants decreased conidia production compared to respective wild-types. The same result was observed in the *PdMfs1* gene of *P. digitatum* [16]. By contrast, the loss of the *Pdmfs2* gene, which is phylogenetically closer to *PdMFS1*, showed significantly altered the yield of conidia [17]. 

Besides, in many cases, eliminating a gene that encodes an MFS protein in pathogenic fungi resulted in the loss of virulence. In this work, infection assays revealed that when citrus fruits were inoculated with the deletant strains(ΔT1 and ΔT2), the disease was developed much slower than when citrus fruits were inoculated with the parental strain Pd1 or with the ectopic transformant ET3, suggesting that *PdMFS1* also plays a role in virulence. A similar result was observed for the *Pdmfs2* MFS transporter in *P. digitatum*, whereas the Δ*Pdmfs2* mutant was less virulent than the wild type [17].

However, the contribution of MFS transporters in virulence remains contradictory. In some cases their involvement in virulence has been demonstrated, as in the case of the *Ctb4* transporter in *C. nicotianae* [10] and the *AaMFS19* transporter in *A. alternata* [15], but does not occur in the case of the *BcMfs1* transporter in *B. cinerea* [9] and the *MgMfs1* transporter in *M. graminicola* [12,13].

Besides, most MFS transporters that affect virulence act as a toxin efflux pumps that contributes to the self-preservation of different toxins, and disruption of these genes drastically reduces pathogenicity [10,37]. However, among the several virulence genes described in *P. digitatum* that included a protein kinase [38], a chitin synthase [39], a o-mannosyl transferase [40], proteases [21], polygalacturonases [41], transcription factors [18,42,43,44], and MAP kinases [45,46], no toxin has been reported for *P. digitatum* to be involved in virulence processes. In that sense, the role of *PdMFS1* in fungal virulence might be related to the elimination of host defense compounds.

Pd149-*PdMFS1* overexpression mutants that raised their infection capacity confirmed the contribution of *PdMFS1* gene in virulence. Additionally, *PdMFS1* was induced during citrus infection in both high virulent and low virulent strains. Comparison of axenic growth and infection, particularly in Pd1, showed that the rate of transcription augmented four times and this reinforces that a *PdMFS1*-based efflux mechanism plays a relevant role in fungal virulence. The effect of *PdMFS1* was previously reported using Northern analysis that showed transcription induction throughout the infection process [21]. 

The mechanisms that control MFS transporters are poorly known. In yeast, MFS transporters are controlled by several stress related transcription factors including Yap1, Msn2, Msn4, and Sfp1 [8], and in *A. alternata*, the transcription of the *AaMFS19* MFS transporter is controlled by the Yap1 transcription activator, Hog1, and Fus3 mitogen-activated protein (MAP) kinases [15]. Currently, it has been reported that the expression of efflux transporters is essentially controlled by fungal transcription factors that belong to the zinc cluster (TFs [Zn_2_Cys_6_]) [5]. The regulation of *P. digitatum* MFS transporters remains unknown, although previous works revealed that two genes involved in signal transduction pathways, *PdSte12* transcription factor and *PdSlt2* (MAP) kinase, could act as negative regulators of *PdMFS1* and other MFS *P. digitatum* transporters [18,46]. Besides, it has been described that fungi regulate and coordinate the different stages of the detoxification system by mutations in particular transcription factors [5]. Another system of MFS transporter regulation is the presence of the insertions in MFS promoters as *Z. tritici* [14] but in this work, no insertions in the promoter region or mutations in the coding region were detected in *PdMFS1.*

In summary, functional characterization of *PdMFS1,* by obtaining gene knockout mutants, revealed that this MFS transporter provides specifically resistance to prochloraz, enables a wide range of fungicide resistance, and contributes to the fungal virulence of this relevant postharvest pathogen of citrus fruit, probably by pumping out toxic compounds from the host and favoring transport of nutrients into fungal cells.

## Supplementary Materials

The following are available online at https://www.mdpi.com/2309-608X/5/4/100/s1, Table S1: Oligo sequence used in this study, Figure S1: Evaluation of virulence of Pd27 overexpression mutants.
