# Supplementary material for: PdMFS1 Transporter Contributes to Penicilliun digitatum Fungicide Resistance and Fungal Virulence during Citrus Fruit Infection
Source: J Fungi (Basel). 2019 Oct 18;5(4):100. doi: 10.3390/jof5040100 (PMC6958471; doi:10.3390/jof5040100)
Supplement: Supplementary file 1 [file jof-05-00100-s001.pdf]

A

B

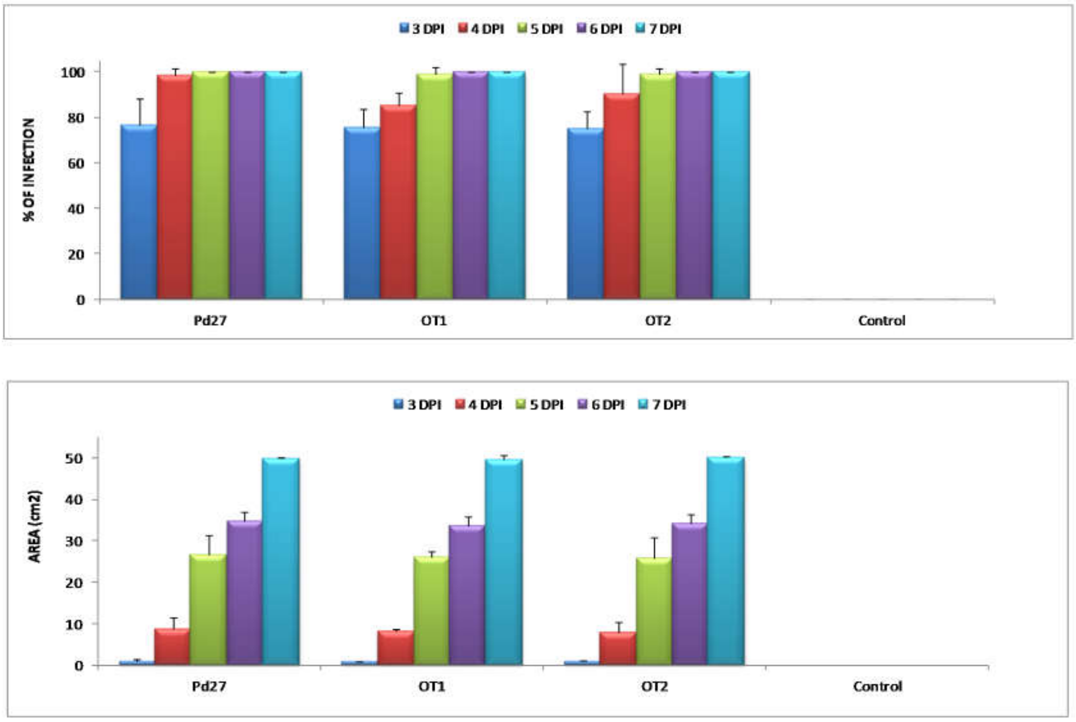

Supplementary Figure S1. Evaluation of virulence as (A) disease intensity (%) and (B) disease severity (cm^2). Virulence evaluation of Pd27 and the overexpression transformants (OT1, OT2). All are mean of three infection experiments. Control correspond to oranges mock inoculated. Error bars represent standard deviation. *Significant differences between treatments using Tukey's test ($P < 0.05$) at each dpi.

Table 1. Oligo sequence used in this study.

Name	Sequence (5′-3′)
M1-1	ACTTTCGGAGTCGGTTGCAC
M1-2	TACCGCCGATGGACAGGAAC
M1-3	ACGAGGAGCTGCTAGGCCGC
M1-4	CTAGGAACGAAGCATTCAGC
M1-5	ATGGAACGCCTTTAACTTGG
M1-6	ATCAAGCAGCAAGTCGTTGG
M1-7	GGTCTTAA**U**ACGAGGAGCTGCTAGGCCGC
M1-8	GGCATTAA**U**CTAGGAACGAAGCATTCAGC
M1-9	GGACTTAA**U**ATGGAACGCCTTTAACTTGG
M1-10	GGGTTTAA**U**ATCAAGCAGCAAGTCGTTGG
M1-11	CTAGATAAGGTTCCCAGGAC
M1-12	GGGTTTAA**U**GCGATAAGAGCCGGATTTCAG
M1-13	GGACTTAA**U**AGCTATTCCATGGACCGATATG
M1-14	ACCTTTGCGAACATATCGGTC
M1-16	AGTCACACCAGCCCCGATAG
HygRt	ATCGAAGCTGAAAGCACGAG
HygFt	GGCAATTTCGATGATGCAGC
HygR	AGCTGCGCCGATGGTTTCTACAA
HygF	GCGCGTCTGCTGCTCCATACAA
hTubF	AGCGGTGACAAGTACGTTCC
hTubR	ACCCTTAGCCCAGTTGTTAC
qTubF	AGCGGTGACAAGTACGTTCC
qTubR	ACCCTTAGCCCAGTTGTTAC
q28SF	TTATAGCCGAGGGTGCAATG
q28SR	TTTCAAGACGGGTCGCTTAC
qH3F	AGGCTCCCCGTAAGCAGCTCGC
qH3R	CGACATGAGGCGGAACTTACCGG
qM1-F	AGTTGCAGCTGCGTACGATG
qM1-R	ACCAAATTGCCGAGACCACG
